# COVID-19 Experiences and Social Distancing: Insights From the Theory of Planned Behavior

**DOI:** 10.1177/08901171211020997

**Published:** 2021-06-02

**Authors:** Rochelle L. Frounfelker, Tara Santavicca, Zhi Yin Li, Diana Miconi, Vivek Venkatesh, Cecile Rousseau

**Affiliations:** 1 Department of Psychiatry, McGill University, Montreal, Quebec, Canada; 2 Department of Epidemiology, Biostatistics, and Occupational Health, McGill University, Montreal, Quebec, Canada; 3 Department of Art Education, Concordia University, Montreal, Quebec, Canada

**Keywords:** health communication, health behavior, COVID-19, discrimination, social distancing

## Abstract

**Purpose:**

The objective of this study is to identify the relationship between COVID-19 experiences, perceived COVID-19 behavioral control, social norms and attitudes, and future intention to follow social distancing guidelines.

**Design:**

This is a cross-sectional study.

**Setting:**

Participants responded to an on-line survey in June 2020.

**Subjects:**

The study included 3,183 residents within Quebec, Canada aged 18 and over.

**Measures:**

Measures include perceived COVID-19 related discrimination, fear of COVID-19 infection, prior exposure to COVID-19, and prior social distancing behavior. Participants self-reported attitudes, perceived behavioral control, and perceived norms related to social distancing. Finally, we measured social distancing behavioral intention.

**Analysis:**

We evaluated a theory of planned behavior (TPB) measurement model of social distancing using confirmatory factor analysis (CFA). The association between COVID-19 perceived discrimination, fear of infection, previous social distancing behavior, exposure to COVID-19, TPB constructs and behavioral intentions to social distance were estimated using SEM path analysis.

**Results:**

TPB constructs were positively associated with intention to follow social distancing guidelines. Fear of COVID-19 infection and prior social distancing behavior were positively associated with behavioral intentions. In contrast, perceived discrimination was negatively associated with the outcome. Associations between fear of COVID-19, perceived COVID-19 discrimination and behavioral intentions were partially mediated by constructs of TPB.

**Conclusions:**

COVID-19 prevention efforts designed to emphasize positive attitudes, perceived control, and social norms around social distancing should carefully balance campaigns that heighten fear of infection along with anti- discrimination messaging.

## Purpose

COVID-19 public health interventions focus predominantly on social and behavioral change strategies to prevent its spread.^1^ The theory of planned behavior (TPB) is a well-established health model used to predict a wide range of health behaviors.^2-5^ TPB hypothesizes a positive relationship between 3 social cognitive factors (attitudes, subjective norms and perceived behavioral control), behavioral intentions, and, ultimately, engagement in health behaviors.^6^ Attitudes refers to the perceived positive and negative outcomes associated with engagement in a health behavior; subjective norms are defined as perceived expectations, values and beliefs of an individual’s social network regarding a health behavior.^6^ Perceived behavioral control encompasses the individual’s perception of the ease or difficulty of engaging in health behavior.^6^

TPB has been used to explain individual-level behavioral intentions and action within the context of infectious diseases.^7-12^ For example, Cheng, Ng^10^ used TPB to understand engagement in prevention activities such as wearing a facemask and washing hands during the SARS epidemic. Emerging research on COVID-19 prevention behaviors is also using the TPB model to explain why individuals do or do not follow social distancing guidelines.^13-15^ Other individual-level factors, such perceived risk, stigma, and personality traits, are theorized as more distal predictors of behavior, with their pathways to behavioral intention mediated by or interacting with TPB constructs.^16^

Specific to COVID-19, distal constructs of disease-related discrimination and perceived risk may be particularly relevant. Discrimination is a feature of stigma, and constitutes unequal treatment on both individual and structural levels with the purpose of maintaining privilege for members of dominant groups at the expense of others.^17^ The COVID-19 epidemic has resulted in social stigma and discrimination against people based on ethnic identities and perceived exposure to the disease.^18^ There is a substantial body of research on the relationship between stigma and health behaviors. Research on infectious diseases such as HIV and tuberculosis, for example, indicates that stigma is associated with decreased help-seeking, disease testing, medication adherence, and disease disclosure (see for example^19,20^). To date, COVID-19 research has examined the relationship between discriminatory attitudes toward people with COVID-19 and engaging in social distancing,^21,22^ and not, to our knowledge, on the association between personal experiences of discrimination and following prevention guidelines.

Risk perceptions include beliefs about vulnerability to danger or harm from a disease and are associated with a wide range of health behaviors.^23-25^ An important component of perceived risk is the level of worry or fear associated with the threat of disease, as this affective aspect of risk may be a strong motivator for engaging in behavior or behavior change.^26^ Research indicates an association between disease-related worry and behavioral intentions,^26,27^ partially mediated by TPB constructs.^28^ Specific to COVID-19, fear of the virus has been associated with following social distancing guidelines.^29-31^

**Figure 1.**
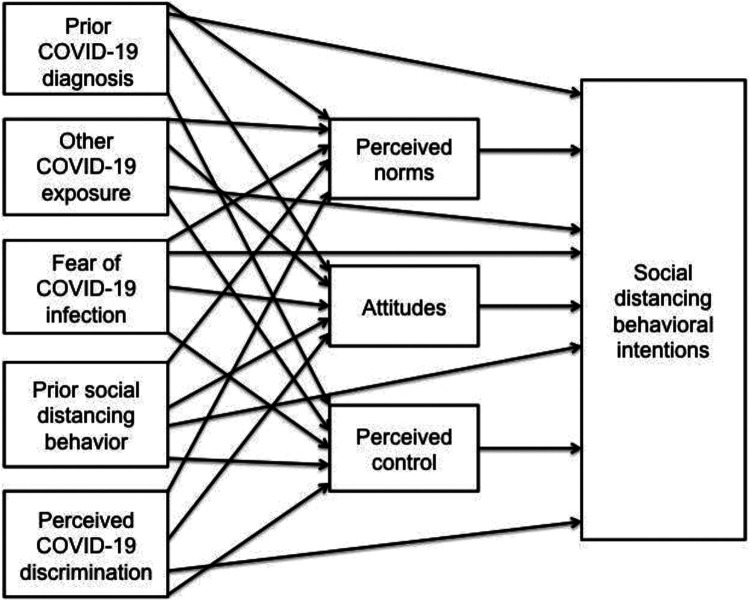
Conceptual model of relationship between COVID-19 experiences, TPB constructs, and behavioral intentions.

The overall objective of this study is to identify the relationship between COVID-19 experiences (including perceived COVID-19 discrimination, fear of infection, prior exposure to COVID-19, and prior social distancing behavior), TPB constructs, and intention to follow social distancing guidelines. We included prior exposure to COVID-19 as an exploratory variable, based on its inclusion in other research on predictors of social distancing.^31^ We use structural equation modeling (SEM) to answer the following research questions: 1) Does the health psychology model of TPB explain social distancing behavioral intentions?; 2) What is the relationship between COVID-19 related experiences and COVID-19 social distancing behavioral intentions?; and 3) Is the relationship between COVID-19 experiences mediated by constructs of TPB? We hypothesized that TPB constructs would be positively associated with social distancing behavioral intentions. Further, we hypothesized that COVID-19 related experiences would be associated with behavioral intentions. Finally, we hypothesized that the relationship between COVID-19 related experiences, and behavioral intentions would be partially mediated by TPB constructs (see Figure 1 for conceptual model).

## Methods

### Design

This is a cross-sectional study.

### Sample

Eligible participants included residents of the province of Quebec, Canada, aged 18 and over. Leger Marketing recruited participants from the Leo panel (a panel of Canadian Internet users), which includes over 400,000 volunteers across Canada, and invited 8,825 individuals who met eligible criteria to complete an on-line survey on COVID-19 via a private link send by email. Participants completed the survey between June 1, 2020 and June 23, 2020 in either English or French, depending upon their preference. A total of 37% of people contacted by Leger (N = 3,273) completed the survey. Individuals with missing data on mediating and outcome variables (n = 90) constituted 2.7% of the sample and were removed from the analysis. Final sample size was 3,183 residents of Quebec. Individuals were given information on the goals of the study, their rights and responsibilities as participants, and provided informed consent prior to starting the survey. Participants received $2 in compensation for their time. This study was approved by the McGill Faculty of Medicine and Health Sciences Institutional Review Board on May 4th 2020. Data were analyzed in 2020.

### Measures

Measures were developed in English and forward translated into French by 2 bilingual members of the research team. Translators discussed and resolved discrepancies using best practices.^32^

*Perceived COVID-19 discrimination.* Participants reported perceived discrimination in the last month because of their presumed COVID-19 status, based on a questionnaire developed by Williams et al (1997)^33^ and adapted to the present health emergency context following a discussion and consensus reached within the research team. Participants were asked: *“Have you been discriminated against because of your presumed COVID-19 status for any of the following reasons in the past month? Check all that apply.”* The list of reasons included (yes/no response format): Age, Gender, Physical Health, Immigration Status, Race/ethnicity, Occupation, Income, Neighborhood you live in. Perceived COVID-19 related discrimination in the past month was measured as a binary variable (yes/no), with individuals who reported 1 or more reason coded as “yes.”

*Prior social distancing.* Prior social distancing behavior was measured on a Likert scale ranging from 1 (None of the time) to 7 (All of the time) in response to the question “*During the past 2 weeks, how often have you kept at least 2 meters distance between yourself and other people who do not live in the same apartment as you when you go out in public?*”

*Fear of COVID-19 infection.* Fear of infection was measured as the sum score of responses to 3 questions ranging from 1 to 7 (sum score range of 3 to 21), with higher scores representing greater fear of COVID-19 infection (α = .92). Questions asked about participant level of fear that someone around them, in their immediate family, or themselves will get sick with COVID-19 in the next month. We included questions related to fear of infection for both the participant and others based on other assessments of worry about infectious diseases^26,27^ and the highly infectious nature of COVID-19.

*Exposure to COVID-19.* Prior COVID-19 diagnosis was measured via 1 question (yes/no) to investigate whether the participant had been diagnosed with COVID-19 in the past month. Other exposure to COVID-19 was measured via 4 questions (also yes/no format) assessing if the participant knew anyone around them, among their neighbors, friends and/or within their household/family who had been diagnosed with COVID-19 in the past 1 month. Responses were categorized into a binary variable (yes/no), with participants who replied yes to at least 1 of the 4 questions considered as having been exposed to COVID-19.

*Theory of planned behavior.* TPB questions were based on a TPB questionnaire developed by Ajzen^34^ and included constructs of attitudes toward social distancing, subjective norms, perceived behavioral control, and intention to social distance. Items were modified to contextualize the constructs with proposed social distancing guidelines as defined by the Quebec government in June 2020. Attitudes toward social distancing (α = .83), perceived behavioral control over social distancing (α = .74), and perceived social norms related to social distancing (α = .79) were each measured as the sum score of responses to 4 Likert scale questions ranging from 1 to 7 (sum score of each subscale ranging from 4 to 28). An example of a perceived behavioral control question is level of agreement with the statement *“Whether or not I practice social distancing on a regular basis for as long as recommended by the Quebec government is completely up to me.”* The measurement of subjective norms included level of agreement with the statement *“Most people whose opinions I value would approve of me practicing social distancing on a regular basis for as long as recommended by the Quebec government.”* An example of attitudes toward social distancing is agreement with the statement *“For me to practice social distancing on a regular basis for as long as recommended by the Quebec government is important.”* Social distancing behavioral intention was measured as the sum score of responses to 3 questions ranging from 1 to 7 (sum score range of 3 to 21), with higher scores indicating greater likelihood of practicing social distancing on a regular basis for as long as recommended by the Quebec government. An example of social distancing behavioral intention is level of agreement with the statement *“I intend to practice social distancing on a regular basis for as long as recommended by the Quebec government.”* Please see supplemental material for a complete list of TPB questions.

*Sociodemographic characteristics.* Self-reported gender, age, household income, physical health, household size, employment status, geographical location and race/ethnicity were included as control variables because of their hypothesized relationship with both independent and outcome variables. Gender was measured as a categorical variable (male, female, other); age was measured as a continuous variable but transformed into categories (18-39, 40-59, 60+). Participants identified their physical health as excellent, very good, good, fair, or poor. Participants reported how many people lived in their household including themselves, which was transformed into a categorical variable of 1, 2, 3,4 or 5 or more people in a household. The categorical variable of employment included responses of unemployed, employed—designated an essential worker, and employed—not designated an essential worker. Household income was a categorical variable (19 k or less, 20-39,999 k, 40-59,999 k, 60-79,999 k, 80-99,999 k, 100 k and over). Geographical location was a binary variable (Greater Montreal Area or elsewhere in Quebec). Race and ethnicity was self-reported as White, East Asian, South Asian, Black, Southeast Asian, Arab, and Other. Please see supplemental material for a correlation matrix of all study variables.

### Analysis

We used univariate statistics to describe the sample and participant responses on all scales. Item-level correlations for all measures can be found in the Online Appendix. We evaluated a 4-factor theory of planned behavior measurement model with confirmatory factor analysis (CFA) using maximum likelihood with a Satorra-Bentler estimation to adjust for the non-normality of the data.^35^ A number of model fit indices were used to measure how well the proposed model fit the study data, including the chi-square statistic, RMSEA, the standardized root mean square residual (SRMR) and the comparative fit index (CFI). Criteria for model fit included: *x*^2^ p > .05, RMSEA < .10, SRMR < .08, CFI > .90.^36,37^ Specific to the individual fit of model components, we assessed factor loadings, with > = .30 used as the desired cut-off value.^10^ Internal consistency reliability of the measurement model was assessed with Cronbach’s alpha, with a desired level of > .70.^38^

We assessed discriminant and convergent validity by estimating the relationships between TPB constructs and external variables theoretically related to the constructs, including fear of COVID-19 and prior social distancing behavior, with Pearson correlation coefficients. We calculated the average variance extracted (AVE) and square root of the AVE for these variables, with the exception of prior social distancing behavior as it was measured by a single item (i.e. not a latent construct). Desired AVE values were > .50, and desired square root AVE values were greater than squared correlations between latent variables.^39^ Authors checked for multicollinearity by calculating variance inflation factor (VIF) values, with values of the relationships between TPB constructs and external variables ranging from 1.13 (fear of COVID-19) to 4.61 (intention), suggesting a moderate yet acceptable correlation between predictors.

We next tested our hypothesis that COVID-19-related experiences would be positively associated with intention to engage in social distancing behavior. We assessed a SEM path analysis model controlling for sociodemographic characteristics, with attitudes, social norms, perceived behavioral control, and intention to engage in social distancing behavior as observed, exogenous variables. We used maximum likelihood estimation adjusted to account for missing data on independent and control variables (command ‘mlmv’). Sensitivity analysis suggested that missing data did not alter the observed patterns of associations. We tested the hypothesis that the association between COVID experiences and the outcome would be partially mediated by perceived behavioral control, social norms, and attitudes by adding in scores on these subscales as observed, mediating variables using bootstrapping (N = 200) to obtain standard errors and confidence intervals.^40^ After establishing a final model, we determined the direct, indirect, and total effects of COVID-19 experiences on behavioral intention scores. Stata 16 was used for all analyzes.^41^

## Results

**Figure 2.**
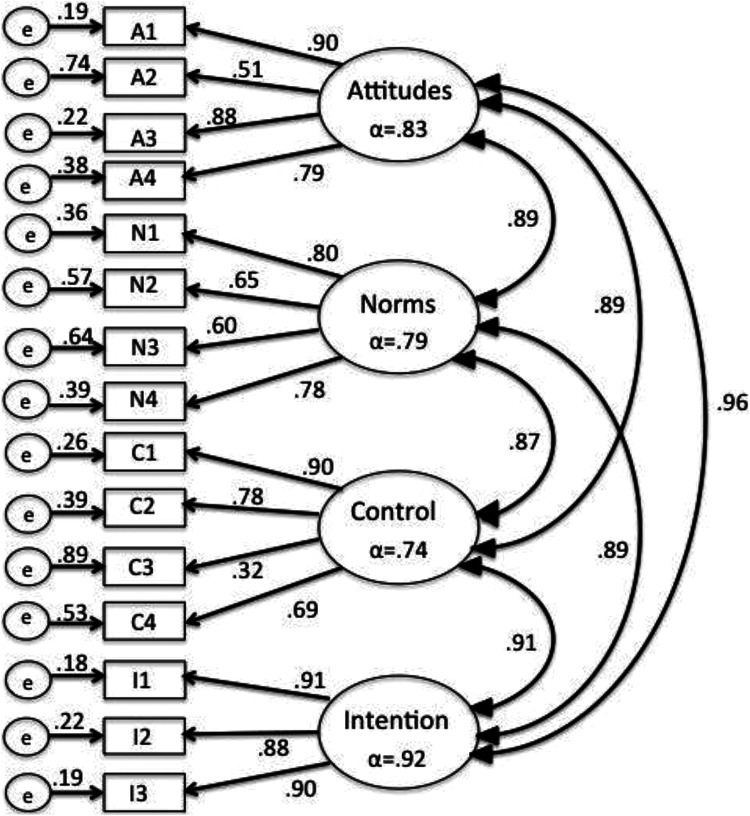
Confirmatory factor analysis of social distancing TPB model (N = 3,183).

Descriptive statistics for study variables can be found in Table 1. CFA indicated that a 4-factor model met desired cut-offs for model fit statistics, with the exception of the chi-square distribution, which was attributed to sample size (*x*^2^ (84) = 688.11, p < .001, RMSEA = .048, CFI = .972, SRMR = .033). All standardized factor loadings were above the desired cut-off of .30, with the lowest loading (.32) item 3 from the Control subscale, “Whether or not I practice social distancing on a regular basis for as long as recommended by the Quebec government is completely up to me.” See Figure 2 for full CFA results.

**Table 1.** Sociodemographic Characteristics of Study Participants in Quebec (N = 3,183).

Variable	n (%)
Gender	
Male	1382 (43.42)
Female	1801 (56.58)
Missing	0
Age	
18-39	1555 (48.85)
40-59	967 (30.38)
60+	661 (20.77)
Missing	0
Race/ethnicity	
White	1573 (49.52)
East Asian	245 (7.70)
South Asian	91 (2.86)
Black	663 (20.83)
Southeast Asian	116 (3.64)
Arab	436 (13.70)
Other	59 (1.85)
Missing	0
Household income	
$19,999 or less	278 (9.72)
Between $20,000 and $39,999	437 (15.29)
Between $40,000 and $59,999	592 (20.71)
Between $60,000 and $79,999	480 (16.79)
Between $80,000 and $99,999	371 (12.98)
$100,000 or more	701 (24.52)
Missing	324
Household size	
1 person	583 (18.73)
2 people	1062 (52.86)
3 people	575 (18.48)
4 people	557 (17.90)
5 or more people	335 (10.76)
Missing	71
Physical health	
Excellent	518 (16.30)
Very good	1077 (33.90)
Good	1021 (32.14)
Fair	446 (14.04)
Poor	115 (3.62)
Missing	6
Employment	
Employed—essential worker	1019 (32.42)
Employed—non essential worker	863 (27.46)
Unemployed	1261 (40.12)
Missing	40
Geographical location	
Greater Montreal region	2114 (68.19)
Outside greater Montreal region	986 (31.81)
Missing	83
COVID-19 discrimination	
Yes	536 (17.27)
No	2572 (82.73)
Missing	75
Prior COVID-19 Diagnosis	
Yes	86 (2.71)
No	2,092 (97.29)
Missing	5
Other COVID-19 exposure	
Yes	888 (28.15)
No	2,266 (71.85)
Missing	29

Internal consistency reliability of TPB constructs was good, with all above the desired cut-off of .70. There were positive correlations between TPB constructs, with Pearson correlation coefficients ranging from .68 (Control and Norms) to .84 (Attitudes and Intentions). Correlations between TPB constructs and constructs of prior social distancing behavior and fear of COVID-19 infection were also positive, as anticipated, with correlations ranging from relatively small (*r* = .18, Fear and Prior behavior) to large (*r* = .58, Prior Behavior and Future Intention). All correlations were statistically significant at p < .01. AVE values indicated good convergent validity, with the exception of Control (.48); square root values indicated problems with discriminant validity, although this is noted as a typical problem among TPB constructs.^42^ See Table 2.

**Table 2.** Pearson Correlations Between Study Constructs.

TPB	Mean (SD)*	Attitudes	Norms	Control	Intention	Fear	Prior behavior	AVE**	√AVE***
Attitudes	5.42 (1.24)	--						.62	.79
Norms	5.59 (1.14)	.73	---					.51	.71
Control	5.38 (1.20)	.75	.68	---				.48	.69
Intention	5.83 (1.31)	.84	.77	.74	--			.81	.90
Fear of infection	4.16 (1.74)	.32	.23	.20	.30	--		.79	.89
Prior behavior	5.68 (1.40)	.53	.55	.51	.58	.18	---	----	---

*Units based on a 7-point Likert scale.

**Average Variance Extracted.

***Square root of Average Variance Extracted.

In path analysis, perceived control (β = .10, SE = .01, 95% CI = .07,.13, p < .001), social norms (β = .22, SE = .01, 95% CI = .19, .25, p < .001), and attitudes about social distancing (β = .39, SE = .02, 95% CI = .36, .43, p < .001) were positively associated with intention to follow social distancing guidelines established by the Quebec government. Fear of COVID-19 infection and prior social distancing behavior were positively associated with perceived control, social norms and attitudes. Perceived COVID-19 discrimination was negatively associated with perceived control and social norms, but not attitudes. Personal diagnosis of COVID-19 was not associated with any of the TPB constructs; other exposure to COVID-19 in the prior month was negatively associated with perceived control (β = −32, SE = .16, 95% CI = −65, −01, p < .05).

In terms of total effects, fear of COVID-19 infection was positively associated with behavioral intentions (β = .16; SE = .01; 95% CI = .13, .18; p < .001), with mediating mechanisms accounting for 79% of the relationship (β = .13; SE = .01, 95% CI = .11, .15; p < .001). Prior social distancing behavior was also associated with social distancing intentions (β = 1.49; SE = .05; 95% CI = 1.39, 1.60; p < .001); a robust 81% of this relationship was attributable to mediating pathways (β = 1.21; SE = .05; 95% CI = 1.12, 1.30; p < .001).

**Figure 3.**
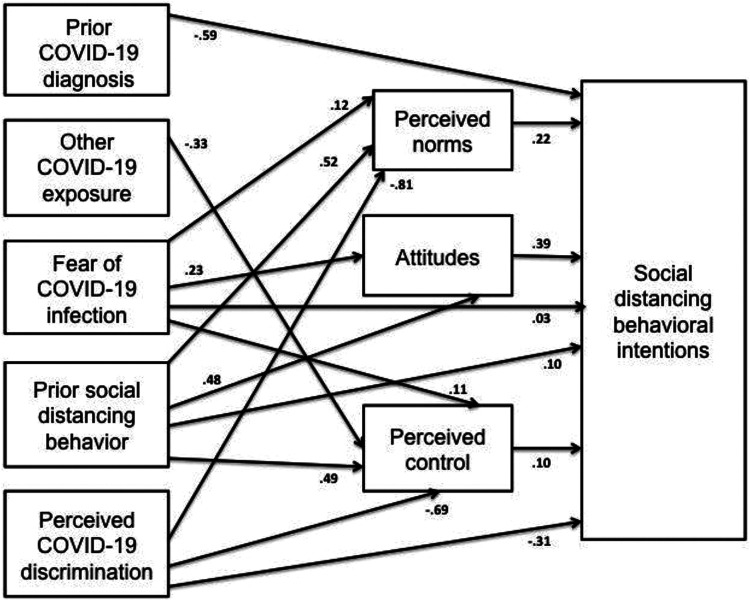
Significant pathways between COVID-19 experiences, TPB constructs, and behavioral intentions. *Covariances between independent variables and mediators are omitted from the diagram for ease of viewing, as are final retained sociodemographic variables of gender, age, physical health and income; Model fit statistics: ×2 (226.16) = p < .001, RMSEA = .051, CFI = .983, SRMR not reported because of missing values.

In contrast, perceived COVID-19 discrimination was negatively associated with the outcome (β −63; SE = .15; 95% CI = −93, −34; p < .001); indirect effects accounted for over half (52%) of this relationship (β = −33; SE = .12, 95% CI = −56, −09; p < .01). There was also a direct, negative association between prior diagnosis of COVID-19 and social distancing intentions (β −59; SE = .30; 95% CI = −1.17, −01; p < .05). There were no significant direct, indirect, or total effects in the relationship between other exposure to the virus and intended social distancing. See Figure 3 for all significant pathways.

## Discussion

This research supports and builds on the nascent body of literature assessing the application of the TPB to COVID-19 prevention behaviors. Other studies have also found a positive association between core TPB constructs and intention to engage in social distancing.^14,15^ A few studies have proposed extended TPB models, adding and finding support for the inclusion of risk perception as an important factor driving behavioral intentions,^13,43^ modeled either as independent of TPB constructs or as a more distal determinant of behavior that is partially mediated by attitudes toward social distancing. We hone in more specifically on the affective dimension of perceived risk, and find fear of COVID-19 is related to behavioral intention both directly and via mediating pathways, the strongest of which is attitudes toward social distancing.

Our findings on the negative association between perceived COVID-19 related discrimination and behavioral intention are to our knowledge unique. Certainly, our findings are aligned with existing research on the negative association between disease-related discrimination and health behaviors related to infectious diseases more generally.^19,20^ However, to date COVID-19 research on disease-based discrimination has focused predominantly on negative consequences of mental health and psychological distress^44,45^ and not behavioral outcomes. This study adds new, critical information on the relationship between discrimination and health behavior by identifying the deleterious impact of these experiences on perceived control over COVID-19 prevention behavior and social norms about social distancing.

Finally, this work provides new insight into if, and in what way, prior exposure to COVID-19 is associated with behavioral intention. One study found no association between prior exposure to the virus (defined as knowing someone diagnosed with or suspected of having COVID-19) and self-reported practice of social distancing.^31^ Our research supports this finding, but indicates that exposure to the virus should be further delineated between having personally had a diagnosis of COVID-19 as opposed to knowing someone else who has tested positive. The negative association between personal diagnosis and behavior intention is not surprising, as individuals who have survived the virus may believe they are immune to future infection and from spreading it to others. It is also noteworthy, in that evidence for long-term immunity to the virus after exposure is still lacking in the scientific community.^46^

Our findings have implications for public health policies and interventions. Based on the TPB, public health interventions should be informed by the goal of promoting positive attitudes toward, and perceived control over, individual-level prevention practice. In addition, it is imperative that interventions instil social norms around recommended behavioral guidelines. Our work indicates that, in pursuit of these goals, it is important to simultaneously maintain a heightened perception of risk of COVID-19 in individuals and communities while integrating anti-discrimination interventions into prevention efforts if we are to be successful in reducing the transmission of COVID-19 locally, regionally, and globally.

We argue that the need to emphasize COVID related risk, and more specifically fear, in health promotion efforts might have unwanted effects on inter-community relations and should be carefully considered. Historically, fear related to disease elicits a quest for meaning in which strangers, or marginalized individuals and communities, are deemed responsible for collective adversity and are scapegoated.^47^ Explanations of COVID-19-related discrimination are informed by social-psychological theories that explore these themes.^48^ Thus in pursuit of instigating fear, we may inadvertently promote discrimination that will have a long-term deleterious effect on promotion of prevention behavior.

Specific to health communication, then, it is necessary to avoid COVID-19 prevention efforts that focus exclusively on fear among vulnerable populations, and instead promote collaboration with affected communities and address larger societal level factors that influence engagement in health behaviors.^47,49^ Our work points to the urgent need for the development, implementation and evaluation of multi-sectoral, community-based anti-discrimination programs which not only improve learning outcomes related to knowledge acquisition (e.g., sourcing the most reliable public health guidelines, improving digital literacy) and cognitive outcomes (e.g., transfer of public health-related learning to health behaviors), but also catalyze concrete outcomes associated with reduction in perpetrating discrimination.

## Limitations

There are several limitations to the internal and external validity of this study. In terms of internal validity, the cross-sectional design prevents us from drawing causal inferences between COVID experiences and behavioral intention. Specific to the measurement model of TPB, we were unable to compare psychometric properties of the scale for each language in which the survey was completed (English and French) and confirm measurement invariance. However, the questionnaire used to assess the TPB model was inspired by well-validated measures, and best practices were followed to ensure the cultural and language validity of translations via the collaboration with bilingual assistants. As noted in the results, while TPB constructs had overall good convergent validity, this was not the case with discriminant validity. Another limitation is the use of path analysis as compared to SEM. Although our decision to focus on observed variables was based on well-established TPB constructs, our results do not account for measurement error as they would if we used a latent variable structural model. With regard to external validity, participants are a convenience sample of Quebec residents drawn from individuals already part of an on-line survey data collection network. As such, our sample is not representative of Quebec residents, and results may not be generalizable to the larger population in Quebec nor other geographical locations in North America. This design also prevents us from evaluating the association between behavioral intention and actual engagement in social distancing. In addition, given that we relied on self-reports we cannot exclude that participants’ responses may have been influenced by a social desirability bias. Nonetheless, online surveys are the safest way to be able to conduct epidemiological research while respecting social distancing during the present pandemic.

## Conclusion

Despite these limitations, this study contributes to COVID-19 public health efforts and the scientific literature. Our work indicates that an extended TPB model is applicable to identifying factors associated with social distancing behavior in the COVID-19 pandemic. In addition to attitudes, social norms and perceived control, perceived COVID-19 related discrimination, fear of infection, prior social distancing behavior, and prior exposure to the virus are associated with intentions to social distance. Perhaps most importantly, our findings are instructive on guiding public health interventions that simultaneously protect people’s lives and develop responses to the epidemic that are inclusive, equitable, and universal.^50^ More specifically, the UN asserts, “discrimination must have no place in our response to the threat it [COVID-19] poses.”^50^ A human rights approach to preventing COVID-19 must not only be inclusive of the overall population, but also include components that actively prevent and mitigate COVID-19 related discrimination.

**So What?**

**What is already known on this topic?**

The Theory of Planned Behavior (TPB) is a well-established health behavior model applicable to research on infectious diseases and health promotion and disease prevention. There is a nascent body of research examining the applicability of TPB to the COVID-19 pandemic and prevention behaviors including wearing masks and social distancing.

**What does this article add?**

An extended TPB model is applicable to identifying factors associated with social distancing behavior in the COVID-19 pandemic. In addition to attitudes, social norms and perceived control, perceived COVID-19 related discrimination, fear of infection, prior social distancing behavior, and prior diagnosis of COVID-19 are associated with intentions to social distance.

**What are the implications for health promotion practice or research?**

COVID-19 health communication efforts should promote collaboration with affected communities and address societal level factors that influence engagement in health behaviors. There is a need for multi-sectorial, community-based anti-discrimination programs that improve outcomes related to knowledge acquisition and cognitive outcomes.

## Supplemental Material

Supplemental Material, sj-docx-1-ahp-10.1177_08901171211020997 - COVID-19 Experiences and Social Distancing: Insights From the Theory of Planned Behavior Click here for additional data file. Supplemental Material, sj-docx-1-ahp-10.1177_08901171211020997 for COVID-19 Experiences and Social Distancing: Insights From the Theory of Planned Behavior by Rochelle L. Frounfelker, Tara Santavicca, Zhi Yin Li, Diana Miconi, Vivek Venkatesh and Cecile Rousseau in American Journal of Health Promotion
