# Supplementary material for: COVID-19 Experiences and Social Distancing: Insights From the Theory of Planned Behavior
Source: Am J Health Promot. 2021 Jun 2;35(8):1095–104. doi: 10.1177/08901171211020997 (PMC8679169; doi:10.1177/08901171211020997)
Supplement: Supplemental Material, sj-docx-1-ahp-10.1177_08901171211020997 - COVID-19 Experiences and Social Distancing: Insights From the Theory of Planned Behavior [file sj-docx-1-ahp-10.1177_08901171211020997.docx]

Supplement 1. Pearson correlation matrix of study variables (N=3,183)

| **Variables** | **1** | **2** | **3** | **4** | **5** | **6** | **7** | **8** | **9** | **10** | **11** | **12** | **13** | **14** | **15** | **16** | **17** |
| --- | --- | --- | --- | --- | --- | --- | --- | --- | --- | --- | --- | --- | --- | --- | --- | --- | --- |
| Diagnosis | 1.00 |  |  |  |  |  |  |  |  |  |  |  |  |  |  |  |  |
| Fear | **.06*** | 1.00 |  |  |  |  |  |  |  |  |  |  |  |  |  |  |  |
| Exposure | **.22*** | **.09*** | 1.00 |  |  |  |  |  |  |  |  |  |  |  |  |  |  |
| Prior behavior | **-.02** | **.18*** | -.03 | 1.00 |  |  |  |  |  |  |  |  |  |  |  |  |  |
| Discrimination | **.21*** | **.11*** | **.17*** | **-.08*** | 1.00 |  |  |  |  |  |  |  |  |  |  |  |  |
| Control | **-.01** | **.20*** | **-.04**** | **.51*** | **-.08*** | 1.00 |  |  |  |  |  |  |  |  |  |  |  |
| Norms | **-.01** | **.23*** | -.002 | **.55*** | **-.09*** | **.68*** | 1.00 |  |  |  |  |  |  |  |  |  |  |
| Attitudes | **-.001** | **.32*** | -.01 | **.53*** | -.03 | **.75*** | **.73*** | 1.00 |  |  |  |  |  |  |  |  |  |
| Intentions | **-.04**** | **.30*** | -.03 | **.58*** | **-.10*** | **.74*** | **.77*** | **.84*** | 1.00 |  |  |  |  |  |  |  |  |
| Employment | **.01** | -.03 | .01 | **-.09*** | -.02 | **-.12*** | **-.11*** | **-.11*** | **-.11*** | 1.00 |  |  |  |  |  |  |  |
| Location | **.05*** | **.12*** | **.15*** | .02 | **.06*** | .01 | **.09*** | **.10*** | **.08*** | .01 | 1.00 |  |  |  |  |  |  |
| Income | **-.03** | **-.10*** | **.06*** | -.02 | **-.13*** | **-.04**** | -.001 | **-.04**** | -.001 | **.28*** | .01 | 1.00 |  |  |  |  |  |
| Physical health | **-.04**** | **.14*** | -.03 | .01 | -.01 | **-.04**** | -.01 | -.01 | .03 | **-.04**** | -.02 | **-.11**** | 1.00 |  |  |  |  |
| Gender | -.03 | **.04**** | .03 | .03 | -.00 | -.01 | **.04**** | .03 | **.06*** | **.04**** | .03 | **.08*** | **.06*** | 1.00 |  |  |  |
| Age | **-.09*** | **-.08*** | **-.14*** | **.23*** | **-.16*** | **.26*** | **.22*** | **.18*** | **.23*** | **-.26*** | **-.09*** | **.04**** | .01 | **-.16*** | 1.00 |  |  |
| Race/ethnicity | **.09*** | **.17*** | **.10*** | -.03 | **.12*** | **-.04**** | -.03 | .03 | -.002 | **.07*** | **.27*** | **-.07*** | **-.04**** | **.04**** | **-.33*** | 1.00 |  |
| Household size | **.03** | **.10*** | **.11*** | **-.04**** | **.05*** | **-.04**** | -.02 | -.004 | -.03 | **.08*** | **.08*** | **.22*** | **-.04**** | **.05**** | **-.29*** | **.25*** | 1.00 |

*p<.01; **p<.05

Supplement 2. CFA unstandardized coefficients and statistics (N=3,183)

| Latent construct | Unstandardized coefficients | z-value | Standard error | p-value |
| --- | --- | --- | --- | --- |
| Attitudes |  |  |  |  |
| A1 | 1 (constrained) |  |  |  |
| A2 | .67 | 33.73 | .02 | <.001 |
| A3 | .98 | 75.53 | .01 | <.001 |
| A4 | .83 | 48.87 | .02 | <.001 |
| Control |  |  |  |  |
| C1 | 1 (constrained) |  |  |  |
| C2 | .95 | 43.79 | .02 | <.001 |
| C3 | .47 | 16.51 | .03 | <.001 |
| C4 | .87 | 44.74 | .02 | <.001 |
| Norms |  |  |  |  |
| N1 | 1 (constrained) |  |  |  |
| N2 | .89 | 31.03 | .03 | <.001 |
| N3 | .80 | 29.63 | .03 | <.001 |
| N4 | 1.09 | 42.02 | .03 | <.001 |
| Intentions |  |  |  |  |
| I1 | 1 (constrained) |  |  |  |
| I2 | .89 | 68.42 | .01 | <.001 |
| I3 | .98 | 80.49 | .01 | <.001 |

**Theory of Planned Behavior Questionnaire**

Social Distancing is defined as remaining out of group settings, avoiding mass gatherings, and maintaining distance (approximately 6 feet or 2 meters) from others when possible.

Perceived Behavioral Control

Use a scale of 1 to 7 to rate your opinion about the following statements regarding social distancing:

1) *For me to practice social distancing on a regular basis for as long as recommended by the Quebec government is*

Impossible 1 2 3 4 5 6 7 Possible

Use a scale of 1 to 7 to rate your opinion about the following statements regarding social distancing:

2) *I am confident that if I wanted to I could practice social distancing on a regular basis for as long as recommended by the Quebec government*

Definitely false 1 2 3 4 5 6 7 Definitely true

Use a scale of 1 to 7 to rate your opinion about the following statements regarding social distancing:

3) *Whether or not I practice social distancing on a regular basis for as long as recommended by the Quebec government is completely up to me*

Strongly disagree 1 2 3 4 5 6 7 Strongly agree

Use a scale of 1 to 7 to rate your opinion about the following statements regarding social distancing:

4) *For me to practice social distancing on a regular basis for as long as recommended by the Quebec government is*

Extremely difficult 1 2 3 4 5 6 7 Extremely easy

Subjective Norms

Use a scale of 1 to 7 to rate your opinion about the following statements regarding social distancing:

1) *Most people whose opinions I value would approve of me practicing social distancing on a regular basis for as long as recommended by the Quebec government*

Strongly disagree 1 2 3 4 5 6 7 Strongly agree

Use a scale of 1 to 7 to rate your opinion about the following statements regarding social distancing:

2) *It is expected of me that I practice social distancing on a regular basis for as long as recommended by the Quebec government*

Definitely false 1 2 3 4 5 6 7 Definitely true

Use a scale of 1 to 7 to rate your opinion about the following statements regarding social distancing:

3) *Most of the people around me practice social distancing on a regular basis*

Definitely false 1 2 3 4 5 6 7 Definitely true

Use a scale of 1 to 7 to rate your opinion about the following statements regarding social distancing:

4) *Most people who are important to me think that I should practice social distancing on a regular basis for as long as recommended by the Quebec government*

Definitely false 1 2 3 4 5 6 7 Definitely true

Attitudes

Use a scale of 1 to 7 to rate your opinion about the following statements regarding social distancing:

1) *For me to practice social distancing on a regular basis for as long as recommended by the Quebec government is important*

Strongly disagree 1 2 3 4 5 6 7 Strongly agree

Use a scale of 1 to 7 to rate your opinion about the following statements regarding social distancing:

2) *For me to practice social distancing on a regular basis for as long as recommended by the Quebec government is*

Extremely unpleasant 1 2 3 4 5 6 7 Extremely pleasant

Use a scale of 1 to 7 to rate your opinion about the following statements regarding social distancing:

3) *For me to practice social distancing on a regular basis for as long as recommended by the Quebec government is*

Extremely worthless 1 2 3 4 5 6 7 Extremely valuable

Use a scale of 1 to 7 to rate your opinion about the following statements regarding social distancing:

4) *For me to practice social distancing on a regular basis for as long as recommended by the Quebec government is*

Extremely bad for public health 1 2 3 4 5 6 7 Extremely good for public health

Behavioral Intention

Use a scale of 1 to 7 to rate your opinion about the following statements regarding social distancing:

1) *I intend to practice social distancing on a regular basis for as long as recommended by the Quebec government*

Strongly disagree 1 2 3 4 5 6 7 Strongly agree

Use a scale of 1 to 7 to rate your opinion about the following statements regarding social distancing:

2) *I will make an effort to practice social distancing on a regular basis for as long as recommended by the Quebec government*

I definitely will not 1 2 3 4 5 6 7 I definitely will

Use a scale of 1 to 7 to rate your opinion about the following statements regarding social distancing:

3) *I plan to practice social distancing on a regular basis for as long as recommended by the Quebec government*

Extremely unlikely 1 2 3 4 5 6 7 Extremely likely
